# New Insights into Osteogenic and Chondrogenic Differentiation of Human Bone Marrow Mesenchymal Stem Cells and Their Potential Clinical Applications for Bone Regeneration in Pediatric Orthopaedics

**DOI:** 10.1155/2013/312501

**Published:** 2013-05-23

**Authors:** Nicola Giuliani, Gina Lisignoli, Marina Magnani, Costantina Racano, Marina Bolzoni, Benedetta Dalla Palma, Angelica Spolzino, Cristina Manferdini, Caterina Abati, Denise Toscani, Andrea Facchini, Franco Aversa

**Affiliations:** ^1^Hematology, Department of Clinical and Experimental Medicine, University of Parma, Via Gramsci 14, 43126 Parma, Italy; ^2^SC Laboratorio di Immunoreumatologia e Rigenerazione Tissutale e Laboratorio RAMSES, Rizzoli Orthopaedic Institute, Via di Barbiano 1/10, 40136 Bologna, Italy; ^3^Paediatric Orthopaedics and Traumatology, Rizzoli Orthopaedic Institute, Via GC Pupilli 1, 40136 Bologna, Italy

## Abstract

Human mesenchymal stem cells (hMSCs) are pluripotent adult stem cells capable of being differentiated into osteoblasts, adipocytes, and chondrocytes. The osteogenic differentiation of hMSCs is regulated either by systemic hormones or by local growth factors able to induce specific intracellular signal pathways that modify the expression and activity of several transcription factors. Runt-related transcription factor 2 (Runx2) and Wnt signaling-related molecules are the major factors critically involved in the osteogenic differentiation process by hMSCs, and SRY-related high-mobility-group (HMG) box transcription factor 9 (SOX9) is involved in the chondrogenic one. hMSCs have generated a great interest in the field of regenerative medicine, particularly in bone regeneration. In this paper, we focused our attention on the molecular mechanisms involved in osteogenic and chondrogenic differentiation of hMSC, and the potential clinical use of hMSCs in osteoarticular pediatric disease characterized by fracture nonunion and pseudarthrosis.

## 1. Introduction

Human mesenchymal stem cells (hMSCs) are pluripotent adult stem cells that can differentiate into different cell types of mesodermic origin, such as osteoblasts, adipocytes, and chondrocytes, as well as into other nonmesodermic cells [1, 2]. MSCs were first discovered by Friedenstein in 1968 as adherent fibroblast-like cells with multipotent differentiation capacities showing that clonal populations belonging to the colony forming unit-fibroblastoids (CFU-Fs) give rise to bone, cartilage, and hematopoietic supportive cells *in vivo* [1, 2]. MSCs have been originally isolated from bone marrow (BM); however, recently other tissues, such as adipose tissue, skeletal muscle, tendon, and trabecular bone, have been identified as potential sources of MSCs [1, 2]. Interestingly, the capacity of vascular pericytes, also known as mural cells that surround endothelial cells and express MSC stem cell markers, has been recently demonstrated in multiple human organs [3, 4]. These cells sustain long-term culture during which they express markers of mesenchymal stem cells and exhibit osteogenic, chondrogenic, and adipogenic potentials [3, 4], thus, supporting the hypothesis of a common perivascular origin of hMSC and postulating the existence of a ubiquitous reserve of multilineage progenitor cells in perivascular cells.

hMSCs were generally defined based on their capacity to self-renew and on the phenotype of their culture amplified progeny because of the lack of a specific and stable cell marker expressed by these cells both *in vivo* and *in vitro*. The International Society of Cellular Therapy (ISCT) proposed the minimal criteria for defining MSCs as follows: (i) adherence to plastic under standard tissue conditions, (ii) the expression of cell surface markers such as CD73, CD90, and CD105 and the lack of expression of CD34, CD45, CD14 or CD11b, CD79 or CD19, and HLA-DR, and (iii) the capacity to differentiate into osteoblasts, adipocytes, and chondroblasts [5]. However, in the last years, other markers have been associated with MSCs such as Stro-1, CD271, vascular cell adhesion molecule (VCAM)-1, and CD146 [6, 7]. Stro-1 is described to be highly specific for BM CFU-F. The function of Stro-1 on MSCs remains unknown, and its expression is downregulated during *in vitro* culture. Stro-1^+^-expanded MSCs were reported to have a better homing capacity, compared to expanded Stro-1^−^ MSCs, suggesting their potential role in MSCs migration and attachment to extracellular matrix [6, 7]. Similarly, CD271 was found in isolated MSCs but down-regulated in culture. CD271 expression could be considered as an early marker of osteogenic capacity although its function remains unknown [6, 7]. Recently, CD271 has been reported to define a subset of MSCs with immunosuppressive and lymphohematopoietic engraftment-promoting properties [6, 7]. Moreover, CD146 expression on MSCs has been associated with pericyte topography. Recently, it has been shown that CD146 expression on MSCs, positive for CD271, correlates with their *in situ* localization [6, 7].

The rationale for the use of MSCs in regenerative medicine is based on the different properties of these cells: (i) ability to migrate to the site of injury, (ii) the potential to differentiate in various mesenchymal tissues and, at least *in vitro*, into different cell lineages, (iii) the ability to release factors that influence cell survival and proliferation, and (iv) the modulation of immune response and inflammation. Beyond their multipotency, the capacity of MSCs to release trophic, anti-inflammatory, and immunomodulatory factors that may limit tissue injury and favor recovery seems to be critical in the proregenerative role of these cells [1, 8]. In fact, several data indicate that MSCs can provide a local microenvironment that supports tissue regeneration such as angiogenesis and osteogenesis through the secretion of several cytokines including epidermal growth factor (HB-EGF), basic fibroblast growth factor (bFGF), platelet-derived growth factor-B chain (PDGF-BB), vascular endothelial growth factor (VEGF), keratinocyte growth factor (KGF), and angiopoietins [9]. All these factors are well known to enhance tissue repair *in vivo* [9]. On the basis of this evidence, hMSCs have generated a great interest in the field of regenerative medicine, particularly in regeneration of bone and cartilage tissues [1, 8]. 

Because the low frequency of MSCs and MSC progenitors in the human BM and other tissues, the *in vivo* use of MSCs may require *ex vivo* expansion to achieve numbers of cells necessary for their clinical applications. Both *ex vivo* expanded and nonexpanded MSCs have been used for bone regeneration [10]. Differences in isolation methods and culture conditions may affect cell yield and the phenotype of the expanded MSCs cells such as reported for the downregulation of STRO-1 and CD271 [6, 7]. The European Group for Blood and Marrow Transplantation (EBMT) has defined a common MSC expansion protocol based on the use of prescreened 10% fetal calf serum (FCS) [11]. Nevertheless, as known, FCS could be theoretically responsible for the transmission of different infections (i.e., zoonoses) or cause immunization in the host recipients. For this reason, serum-free conditions have been investigated as well as the use of both autologous and allogeneic serums [10]. It has been reported that autologous serum was superior in terms of capacity of expansion of MSCs as compared to both allogenic serum and FCS [10]. Recently, platelet lysate has been demonstrated to be a useful substitute for FCS in MSC expansion [10]. 

Cultures of MSCs show heterogeneity, differential growth rate, and developmental potentials exhibited by individually expanded MSC clones. As a consequence, researchers are actively attempting to determine the genotypic and proteomic profiles of long-lived MSC clones in order to elucidate the mechanisms that regulate and maintain primitive MSC populations in expanded cells. Little is known regarding the proportion of expanded MSCs that remain as multipotential stem cells used in the cell therapies. Moreover, the efficacy of the MSCs in tissue regeneration largely depends on their homing capacity and the microenvironment that are critical in limiting or expanding *in vivo* the differentiation capacity of these cells [12]. 

**Figure 1:**
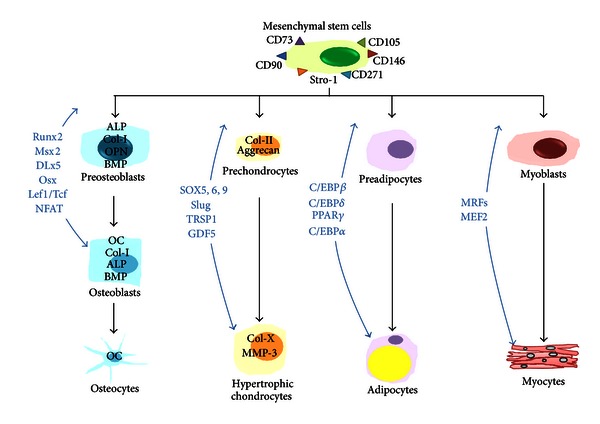
Multipotent differentiation of BM hMSCs. The commitment and differentiation of hMSCs towards osteogenic, chondrogenic, adipogenic, or myogenic lineage are regulated by specific transcription factors (indicated in blue). ALP, alkaline phosphatase; Col-I, type I collagen; OPN, osteopontin; BMP, bone matrix protein; OC, osteocalcin; Col-II, type II collagen; Col-X, type X collagen; MMP-3, matrix metalloproteinase-3.

In the past few years, the molecular mechanisms involved in the differentiation process of hMSCs have been elucidated, and the transcription factors involved in these processes identified that (Figure 1) these new acquisitions could improve our future expansion strategies and clinical use of MSCs. In this paper, we focus our attention on the molecular mechanisms involved in osteogenic and chondrogenic differentiation of hMSC, and the potential clinical use of hMSCs in osteoarticular pediatric disease characterized by fracture nonunion and pseudarthrosis.

## 2. Osteogenic Differentiation of Bone Marrow hMSCs

The osteogenic differentiation of BM hMSCs is regulated either by systemic hormones, such as parathyroid hormone (PTH), estrogens, and glucocorticoids, or by local growth factors, including the bone morphogenetic protein (BMP) family, transforming growth factor-beta (TGF-**β**), and fibroblast growth factor-2 (FGF-2) [13]. These factors activate specific intracellular signal pathways that modify the expression and activity of several transcription factors in hMSCs, which result in osteoblastic differentiation rather than chondrocytic, adipocytic, or myogenic ones [13] (Figure 1). 

Runt-related transcription factor 2 (Runx2), also named Cbfa1 or AML3, is the major transcription factor regulating osteoblast commitment and osteogenic differentiation of MSCs [13, 14]. Studies on mice lacking Runx2 indicated that the expression of Runx2 is critical for MSC differentiation toward the osteoblast lineage. Runx2-deficient (Runx2^−*⁄*−^) mice completely lack osteoblasts and bone formation, demonstrating a pivotal role for this factor in osteoblastogenesis [13, 14]. However, Runx2 overexpression also impairs bone formation in mice [15], indicating that its effect is dependent on the stage of osteoblast differentiation. Runx2-overexpressing mice also show enhanced bone resorption, possibly through increased expression of the osteoclast stimulating factor receptor activator of nuclear factor **κ**B ligand (RANKL) by osteoprogenitor cells [16]. Activation of Runx2 in human BM MSCs induces expression of the osteoblast-related markers collagen I, alkaline phosphatase, and osteocalcin during the early stages of osteoblast maturation [16, 17]. Both expression and activity of Runx2 are tightly regulated by other transcription factors as well as protein-DNA or protein-protein interactions. Runx2 itself is regulated by phosphorylation of the extracellular signal-regulated kinase (ERK)/mitogen-activated protein kinase (MAPK) pathway [18]. Hey-1, Hoxa2, Stat1, and Sox9 interact with Runx2 and inhibit its expression and/or transcriptional activity and thus are negative regulators of osteoblast differentiation. Runx2 activity is also positively regulated by transcription factors such as TAZ, Hoxa10, or BAPX-1 [13, 14]. Moreover, multiple signaling pathways converge to interact with Runx2 to regulate osteoblast differentiation, including binding with activator protein 1 (AP-1) and activating transcription factor 4 (ATF4) which, with Runx2, regulate osteocalcin and osterix (Osx) [13, 14]. Osx-deficient mice lack osteoblast formation, and Osx acts downstream of Runx2 [13, 14]. In mouse systems, osteogenic factors stimulate osteogenesis through regulation of these transcription factors. BMP-2 promotes Runx2 and Osx expression in murine osteoprogenitor and osteoblastic cells, and TGF-**β** and FGF-2 may enhance osteoblast differentiation by increasing Runx2 expression and activity [13, 14].

Wnt signaling plays a critical role in the regulation of osteoblast formation [19–23]. Canonical Wnt signaling pathway is activated by Wnt 1/3a that triggers phosphorylation of GSK3/Axin complex, leading to stabilization and nuclear translocation of the active dephosphorylated (dephospho) **β**-catenin, which in turn activates the lymphoid enhancer factor-1/T-cell factor transcription system [19–23]. BMP-2 and other osteogenic molecules induce osteoblastic differentiation of murine MSCs by stimulating Wnt signaling through modulation of Wnt stimulators and/or inhibitors [23]. Some reports show that BMP-2 inhibits Wnt signaling [24]. 

Several molecules negatively regulate canonical Wnt signaling by inducing phosphorylation and subsequent degradation of **β**-catenin, inhibiting osteoblast differentiation in murine osteoprogenitor cells. Dickkopfs (DKKs) including DKK-1 [25], the secreted frizzled-related proteins (sFRPs), such as sFRP-1, -2, -3, -4, and Wnt inhibitory factor-1 are the major soluble Wnt inhibitors present in murine osteoblasts, which block early osteoblast formation and induce the death of immature cells [26, 27]. *In vivo* models support the role of canonical Wnt signaling in the regulation of bone formation [28–30]. Inactivating mutations of the LRP5 Wnt coreceptor cause osteoporosis, indicating that Wnt-mediated signaling via LRP5 affects bone accrual during growth and is important for the establishment of peak bone mass. Constitutive activating LRP5 mutations impair the action of normal antagonists of the Wnt pathway such as DKK-1 and increase Wnt signaling, which result in high bone density [29, 30]. Moreover, a targeted destruction of LRP5 in mice induces a low bone mass phenotype due to decrease of osteoblast proliferation and function independently to Runx2 as demonstrated by a normal Runx2 expression in Lrp5^−*⁄*−^ mice [31]. On the other hand, it has been reported that canonical Wnt signaling promotes osteogenesis by directly stimulating Runx2 gene expression [32]. In sFRP-1 null mice, which exhibit activated Wnt signaling and high bone mass phenotype, there is a significant increase of both T-cell factor and Runx2 expression [32]. Similarly, overexpression of the canonical activator Wnt10b in mice protects from bone loss of estrogen deficiency and shifts MSCs toward osteoblastic lineage by induction of Runx2 [33].

Despite the consistent findings between human genetic and mouse studies, which indicate that activation of canonical Wnt signaling stimulates bone formation, data obtained from human MSCs indicate that canonical Wnt activation by Wnt3a in human BM hMSCs suppresses osteogenic differentiation rather than stimulates osteoblastogenesis [34–37]. These results suggest that Wnt signaling is required to maintain hMSCs in an undifferentiated state. It is likely that the effect of canonical Wnt signaling on osteogenesis in hMSCs depends on the level of Wnt activity given that hyperactivation of Wnt signaling by overexpressing LRP5 [29] can enhance osteogenesis, whereas exogenous levels of Wnt3a inhibit osteoblast differentiation [34, 35]. On the other hand, the effect of canonical Wnt signaling may depend on the stage of differentiation of the cells. Accordingly, it has been reported that canonical Wnt signaling antagonizes the terminal steps of osteogenic differentiation as demonstrated by the evidence that mice lacking DKK-2 are osteopenic [38]. Consistently, an increased expression of Wnt antagonists during late osteoblast differentiation has been shown [39, 40].

Together with canonical Wnt signaling, a noncanonical Wnt pathway independent to the activation of **β**-catenin has been extensively demonstrated [19, 21, 41, 42]. Noncanonical Wnt signals are transduced through frizzled (FZD) receptor and Ror2 coreceptor to several cascades involving disheveled or Ca^++^-dependent pathways. Rho family of small GTPase (RhoA, Rac) and JNK are downstream effectors of disheveled. Nemo-like kinase and the nuclear factor of activated T cells (NFATc1) are Ca^++^ effectors of noncanonical pathways [41, 42]. Wnt-4, -5, and -11 proteins have been identified as specific activators of noncanonical Wnt pathway [41, 42]. Wnt5a is one of the most highly investigated noncanonical Wnt and is involved in almost all aspects of noncanonical Wnt signaling [43]. The noncanonical Wnts can trigger intracellular calcium flux, which can lead to the activation of calcium-dependent signaling molecules such as calmodulin-dependent protein kinase II (CAMKII) and protein kinase C (PKC). In addition to FZD receptors, Wnt5a can also bind and activate the Ror2 receptor, resulting in the activation of the actin-binding protein filamin A and JNK signaling pathway. Wnt5a is also capable of inhibiting the activation of canonical signaling pathway by numerous mechanisms, either by calcium signaling through CAMKII or through Ror2 signaling pathway. In turn, this pathway can stimulate the TAK1-NLK pathway to phosphorylate and inactivate the active **β**-catenin transcription complex [43]. Recent evidence suggests that noncanonical Wnt pathway activation by Wnt5a blunts the inhibitory effect of Wnt3a on osteogenic differentiation of hMSCs and stimulates osteoblast differentiation [44, 45]. Similarly, noncanonical Wnt4 signaling enhances bone regeneration of hMSCs *in vivo* [44]. The proosteogenic effect of Wnt5a could be mediated by activation and homodimerization of the receptor Ror2 in hMSCs, whereas Wnt3a has no effect on Ror2 activation and homodimerization [45, 46]. It has been consistently shown that Ror2 overexpression in hMSC induces expression of the osteogenic transcription factors Osx and Runx2 and induces osteogenic differentiation [46–48]. Finally, it has been recently demonstrated that noncanonical Wnt pathway through Nemo-like kinase represses peroxisome proliferation activated receptor-**γ** transactivation and induces Runx2 expression promoting osteoblastogenesis in BM MSCs suggesting a potential relationship between noncanonical pathway and Runx2 activity [49]. This evidence indicates that noncanonical Wnt signaling has a critical role in the osteogenic differentiation process in hMSCs and that modulation of noncanonical Wnt signaling could be used to increase bone formation. 

## 3. Chondrogenic Differentiation of hMSCs

Different MSC sources (BM, adipose tissue, synovial tissue, umbilical cord blood, periosteum, and synovial membrane) have been studied [50–52] to establish their chondrogenic potential, and BM and adipose tissue sources were the most studied for historical and easy accessibility reasons. Different parameters were considered and studied in hMSCs chondrogenic differentiation. In particular, hMSCs culture condition was one of the steps analyzed and was evidenced that hMSC expansion *in vitro* required FGF-2 to enhance the proliferative and chondrogenic potential [53, 54]. Different studies demonstrated that the potential of hMSCs chondrogenic differentiation was enhanced using serum-free media [55], cells at passages between 3 and 6 [56], culture in three dimensions (i.e., micromasses) [57], incubator with low-oxygen tension (2–5% O_2_) [58], and mechano stimulation [59].

Chondrogenic differentiation of BM hMSCs has been widely studied *in vitro* in micromass pellet condition that favors the induction of the first phase, characterized by cell condensation, as well as cell-cell and cell-extracellular matrix (ECM) interactions [55, 56]. Subsequently, cells progress into a highly proliferative phase and start to produce typical components of the cartilaginous matrix (i.e., collagen type 2, collagen type 9, aggrecan, link protein, cartilage oligomeric matrix protein, and matrilin 1) [60–64]. Finally, cells become progressively rounded similar to chondroblast and then undergo hypertrophy expressing collagen type X and MMP13. 

The different stages of chondrogenic differentiation are regulated by signaling factors like BMPs, FGF, TGF-**β**, Wnt, and Indian hedgehog (Ihh) that promote these processes. Different key transcription factors (SOX9, SOX5, SOX6, Slug, TRSP1, and GDF5) [65–68] have a crucial role in the control of stem cells properties as well as in the chondrogenic commitment status by driving the mesenchymal condensation and differentiation [65–67]. 

SOX9 is considered the master chondrogenic transcription factor since its expression is elevated in condensing mesenchymal progenitors [69, 70]. SOX9 specifically interacts with two binding sites for HMG-domain proteins and activates elements in the promoters of Col2a1, Col9a1, Col11a2, and aggrecan. Removal of SOX9 before mesenchymal condensation results in mice without cartilage development, while loss of SOX9 after condensation causes the arrest of chondrocytes differentiation [71]. SOX9 markedly inhibited activation of Wnt/**β**-catenin-dependent promoters and stimulated degradation of **β**-catenin [72] by directing intracellular degradation, thus, favoring chondrocyte differentiation and delaying hypertrophic chondrocytes differentiation [69]. Studies performed on embryos in different stages of gestation evidenced that SOX9 and Runx2 control the transition from prehypertrophic to hypertrophic chondrocytes [73]. It has been demonstrated that upregulation of type X collagen (col10a1), typical hypertrophic marker, is also regulated by overexpression of Wnt8c, Wnt9a and **β**-catenin that inhibited SOX9 and type II collagen (col2a1) and induced Runx2 trascription factor [74]. SOX9 and Runx2 physically interact in MSC and while SOX9 can inhibit Runx2 transactivation, on the other hand, Runx2 exerts reciprocal inhibition on SOX9 transactivity [72]. These data provide convincing evidence that Runx2 induces chondrocyte hypertrophy. Rescue of Runx2 expression in mesenchymal condensation of Runx2-null mice restores chondrocytes hypertrophy without affecting osteogenesis [75].

SOX9 activates SOX5 and SOX6, and together they promote the early stage of differentiation when chondroblast proliferates and forms columnar chondrocytes and suppresses their terminal stage [76]. SOX5/SOX6 double-knockout mice show evidence of a severe, generalized chondrodysplasia indicating redundant functions of both transcriptor factors in chondrogenic differentiation [77]. SOX9 is not dependent on SOX5 and SOX6, and its level remains unaffected in mice with loss of SOX5 and SOX6. SOX5 and SOX6 have been suggested to potentiate transactivation of chondrocyte-specific gene SOX9 through cooperative binding to multiple recognition sites on enhancers [78]. SOX9, SOX5, and SOX6, often referred as SOX Trio, exert a cooperatively combined action to further upregulate the expression of type II, IX, and XI collagens and aggrecan [79]. Recent data have demonstrated that SOX5 and SOX6 proteins enhance miR-140 microRNA by promoting transactivation ability of SOX9 as homodimer, thus, providing new insights into cartilage-specific gene regulation by SOX trio [80].

## 4. Potential Clinical Use of hMSCs for Bone and Cartilage Regeneration in Pediatric Orthopaedics

The regenerative medicine approaches have been extensively studied to improve bone healing. Fracture nonunion and pseudarthrosis, defined as a false joint associated with abnormal movement at the site, are the prevalent bone pathology conditions where filling of the space with bone tissue is needed. However, in these conditions, bone supply is limited as well as autologous bone harvesting is accompanied with high rates of morbidity and allogeneic transplantation that inhere the risk of rejection [81]. Presently, hMSCs seem to be the most promising candidates for bone regeneration due to their osteogenic differentiation capacity [81]. 

The main rationale for the use of hMSCs in bone repair is based on the direct ability to generate osteoprogenitor cells by hMSCs further enhanced by the use of bone-stimulating agents such as BMP-2 also as genes delivered into hMSCs by viral or nonviral delivery systems [6]. However, more recent evidence suggests that the potential clinical effect of hMSCs is not only due to the direct conversion into osteoblasts, but also to an indirect one referred as paracrine effects supported by the production of several cytokines involved in osteoblastogenesis including bFGF, VEGF, PDGF, and TGF-**β** [6]. In line with this hypothesis, it is possible that hMSCs can act to orchestrate the host response, increasing vascularization and host cell migration including osteoblastic cells. 

The potential clinical use of hMSCs in fracture healing has been supposed by Hernigou et al. [82] who treated patients with atrophic nonunion of the tibia with percutaneous injection of concentrated BM. Others reported the use of scaffold loaded with expanded autologous hMSCs [83]. Scaffolds are used as carriers for expanded MSCs before bone implantation. Scaffolds mimic the natural environment of the bone matrix. The combined effect of scaffolds with growth factors has been shown to increase the formation and vascularization of bone [84–86]. Several scaffolds have been investigated in preclinical and clinical studies, although hydroxyapatite (HA) and calcium phosphate seem to be more useful because of their excellent osteoconductive properties [84–86]. HA provides good strength but is not resorbed, while beta-tricalcium phosphate (*β*-TCP) is fragile but has a greater capacity for resorption. Hence, a combination of HA and *β*-TCP, biphasic calcium phosphate, is generally used [84–86]. Moreover, HA can be combined with biodegradable polymer/bioceramic composites, including polylactic-co-glycolic acid. Three-dimensional polymer scaffolds with dimensions of 150–500 *μ*m have been shown to have excellent stability and used for MSC therapy [84–86]. Expanded MSCs for three weeks and seeded them on macroporous HA scaffolds have been used to treat fracture nonunion [85]. 

Although the combination of growth factors and scaffolds remains a promising approach, genetic modification of MSCs to express growth or transcription factors, which involves either transfection of MSCs through viral vectors or by the use of nonviral vectors, is a suitable alternative. Viral vectors have been demonstrated to be a better delivery as compared to nonviral vectors for the higher efficiency in transfection and expression of the osteogenic factor [86, 87]. BMP-2-transfected MSCs induce bone formation in mouse and in bony union of mouse radial defects. In another study, BM-derived stem cells, which had been genetically modified with BMP4, were used to repair defects in the calvarial bone in rabbits [86, 87]. Moreover, permanent expression of BMP4 by retrovirus in BMSCs leads to regeneration of calvarial defects in rats [86, 87]. Finally, overexpression of Runx2 has been demonstrated to stimulate osteoblast differentiation of engineered MSCs in mice [86, 87]. However, to date, any clinical studies have not applied *ex vivo*-expanded genetically modified MSCs because of the need to identify the optimal vector to ensure effective, safe, and consistent treatment.

Both autologous and allogeneic MSCs are potentially used for bone regeneration [88]. The use of allogeneic MSC for repair of large defects may be an alternative to autologous and allogeneic tissue-grafting procedures. Several authors have reported near equivalency when comparing allogeneic to autologous or syngeneic MSCs in healing models [88]. An allogeneic approach would enable MSC to be isolated from any donor, expanded and cryopreserved, providing a readily available source of progenitors for cell replacement therapy. Their immunomodulatory properties have raised the possibility of establishing allogeneic MSC banks for tissue regeneration. However, actually whether autologous or allogeneic MSCs should be preferred in the setting of regenerative medicine needs to be further investigated.

Congenital pseudarthrosis refers to a spontaneous fracture, which progresses to nonunion. In pediatrics, congenital pseudarthrosis is one of the most frustrating conditions encountered in orthopaedic surgery because of the difficulty in achieving healing. In fact, the most commonly used methods of treatment are the different modifications of Ilizarov technique, vascularized fibular grafting, bone grafting with intramedullary fixation, and Boyd's double-bone grafting. Finally, in case of bad results, significant shortening of the leg, even amputation, has to be considered. Numerous treatment options have been explored with varying levels of success [89, 90]. Recently, a combined surgical technique using autologous BM hMSCs as adjuvant to the surgical stabilization by an external fixator or an intramedullary nailing was reported [91]. These combined techniques in tibia congenital pseudarthrosis were used in children with and without neurofibromatosis [91]. Neurofibromatosis type I (NF1) is one of the most common congenital diseases with a prevalence of one in 3000–4000 [92]. Neurofibromas, café-au-lait spots, and Lisch nodules are the hallmark of the disease [92]. However, NF1 is also characterized by several skeletal manifestations including congenital pseudarthrosis of the tibia. Bone alterations are likely to be related to a deficient osteoblast function with enhanced osteoclast activity and survival occurring in approximately 2-3% of children suffering from NF1. An impairment of osteogenic differentiation by MSCs has been demonstrated in NF1 [93, 94]. Mouse models lacking both alleles of NF1 specifically in limb osteochondroprogenitors (*Nf1*Prx model) and mature osteoblasts (NF1_Ob_^−/−^ model) displayed bone abnormalities that demonstrated the existence of a cell-autonomous role of NF1 in the mesenchymal lineage [95]. Defects in osteoblasts have also demonstrated that dysfunctions caused by loss of NF1 in osteoblasts impair callus maturation and weaken callus mechanical properties [96]. The most affected bone is the tibial diaphysis and dysplastic lesion having the radiological appearance of an anterior bowing, or a sclerotic lesion, or a fracture [97]. The congenital pseudarthrosis of the tibia (CPT) with/without NF1 is one of the most complex and disabling pediatric skeletal diseases. Congenital pseudarthrosis of the tibia appears to be caused by fibrous hamartoma originating from aberrant growth of NF1 haploinsufficient periosteal cells, which failed in terminal osteoblastic differentiation and was arrested at a certain stage of this process involving Runx2 and Wnt signaling [98]. Conservative treatment includes bracing of the affected limb continued until the end of skeletal growth. Surgical treatment includes several procedures, which are resection of the nonunion and reconstruction with either autologous nonvascularized bone grafting and intramedullary fixation or one-stage shortening followed by an Ilizarov external fixator to perform lengthening [99, 100]. All these procedures have frequently unsatisfactory results because of the need for several operations for recalcitrant nonunion, residual deformity, and limb-length discrepancies [101]. Recently, the use of autologous hMSCs has garnered great interest, because their biological properties candidate them as a suitable source of osteogenic precursor for bone repair and regeneration, both in cell therapy and tissue engineering applications. Surgical treatment of pseudarthrotic lesions could benefit from regenerative medicine, which provides a biological approach to consolidation, based on the use of hMSCs and growth factors. These elements could lead to tissue healing and consolidation, in cases where traditional techniques fail. The biological bases of healing show that hMSCs transplantation derived from BM resident in iliac crest (IC) was able to differentiate into osteoblast with higher efficacy compared to resident in proximity of the pseudarthrosis site [91]. In our study, all the healing patients are NF1^+^, and, in these patients, we found that osteogenic potential hMSCs derived from the iliac crest were more than those derived from the affected site [91]. Leskelä et al. [93] also confirmed these results. However, on the feasibility of this approach, any data are still not available on the osteogenic potential of MSC transplanted, because the microenvironment properties could also impair the successful procedure. Moreover, platelet-rich plasma (PRP) has been shown to stimulate osteoblast proliferation *in vitro* [102], probably due to the high levels of growth factors that can improve the BM hMSCs to form osteogenic and angiogenic tissue. The known growth factors include PDGF, TGF-*β*1 and *β*2, and IGF-1. Other growth factors present in PRP are VEGF and EGF [103].

**Figure 2:**
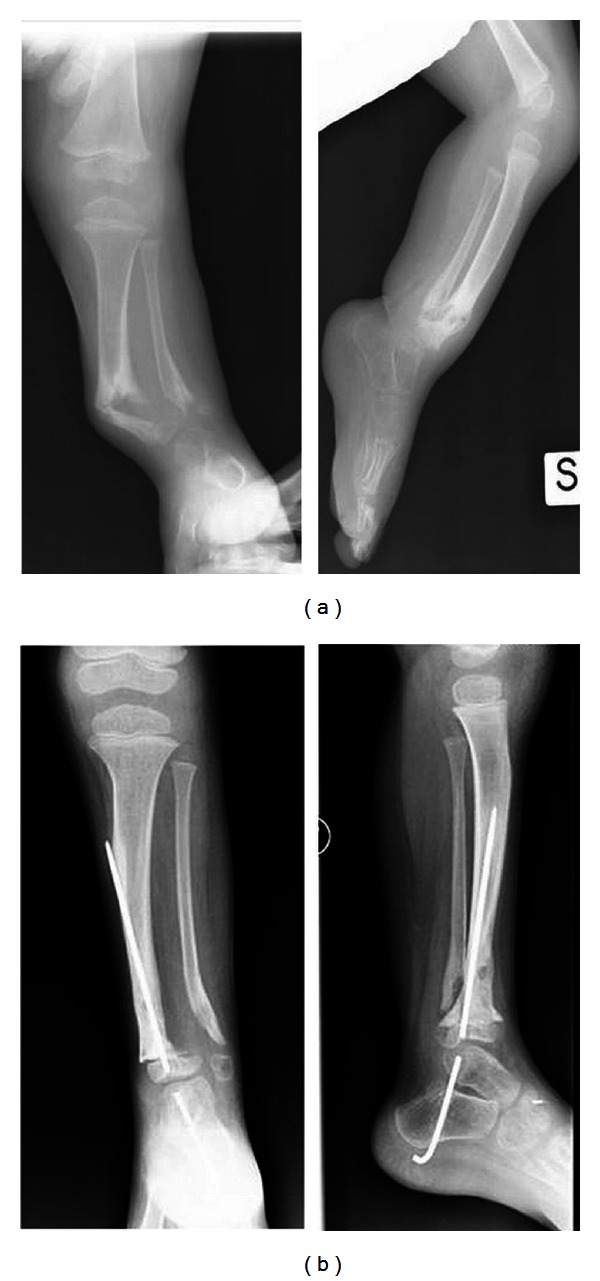
Clinical effect of the use of combined BM hMSC-based technique. Radiographics of a representative patient affected by NF1 with a tibial pseudarthrosis before (a) and three months (b) after the use of autologous BM hMSCs combined with platelet-rich fibrin and lyophilized bone.

In our study, 10 patients affected by refractory CPT were treated by using hMSCs derived from the iliac crest (IC-MSCs), PRF, and lyophilized bone. In six patients, CPT was associated with NF1. Any complications related to marrow donor site were reported in this group of patients [91]. Bone consolidation was obtained in three patients who had CPT and NF1. In these patients, the IC-MSCs exposed to autologous serum were able to form mineral nodules *in vitro*, while the mineralizing ability was totally abrogated in patients with a poor clinical outcome [91]. Interestingly, we also reported that bFGF serum levels were significantly lower in patients who did not heal after surgery. The relationship between bFGF and bone healing was supported by *in vitro* experiments. In patients with poor clinical outcome, autologous serum was not sufficient to induce *in vitro* mineralization; however, it did occur when cells were cultured with bFGF [104]. The enhanced bone formation might indirectly depend on vascularization; in fact, the entirely healed patient had distal tibial pseudarthrosis. The distal tibia has naturally a better vascularization than the proximal site. An increased vascularization was also achieved by exogenous delivery of angiogenic growth factor by platelets, and hMSCs implant contributed to improve bone formation and consolidation. The improved vascularity might provide better nutrition and increase resorption and substitution by healthy tissue [105]. It is likely that this kind of therapy has a good clinical application because both components are autologous and easy to perform; it could reduce the healing time and the number of surgical treatments, especially in children, and the use of this combined technique may induce early bone healing and preserve the articular stiffness due to several surgical procedures and also reduce the leg's discrepancy that is due to the repetitive fractures. For all these evidences, it is reasonable to use this technique in patients treated with several operations before a demolitive surgery or a much more aggressive surgery like vascularized fibula.  Figure 2 shows a representative patient with tibial pseudarthrosis healed after this type of combined treatment for three months. 

Osteogenesis imperfecta (OI) is a genetic disorder of MSC due to mutations in the COL1A1 and COL1A2 genes characterized by production of defective type I collagen, generalized osteopenia that leads to bone deformities and fractures in children [106]. On the contrary, pseudarthrosis was not reported in this type of patients. Several pre-clinical studies in mouse indicate a role for cell therapy with MSCs in the treatment of OI [84, 85, 107–110]. Allogeneic BM transplantation has been reported in children suffering from severe OI and reported increased growth velocity, total body mineral content, and fewer fractures [108]. In a later study, isolated MSC from the original BM donors was gene marked and infused into six patients [109]. A low level of donor MSC was detected by PCR, and growth velocity was accelerated. The results, therefore, suggested the safety and feasibility of MSC therapy and a potential benefit to children with OI. Moreover, it has been reported that allogeneic HLA-mismatched fetal liver MSCs uterus transplantation in a fetus with severe OI is capable of engrafting and differentiating into bone [110].

Cartilage is vulnerable to injury and has poor potential for repair. However, unlike bone, cartilage regeneration remains elusive. Procedures devoted to recruit stem cells from BM by penetration of the subchondral bone have been widely used to treat localized cartilage defects [111–113]. More recently, autologous chondrocyte implantation, alone or in combination with MSCs, has been introduced [111]. The use of MSCs in cartilage regeneration includes microfracture, implantation, and recruitment from the synovial membrane [112, 113]. However, all these applications are based on very small case series. The feasibility, efficacy, and safety of autologous MSC implantation for the treatment of cartilage defects were reported in osteochondral defect animal models demonstrating that MSCs transplanted into full-thickness cartilage defects recreate the layered arrangement of articular cartilage [84, 85, 113]. In human studies it has been reported that the patients that received autologous MSC therapy demonstrated substantial improvement in their symptoms, showing evidence of cartilage repair and the ability to resume their sporting and daily activities [84, 85, 113]. The progress of the patients was followed for up to 5 years, with no evidence of adverse effects from the MSC therapy [84, 85, 113]. The intra-articular injection of MSCs has been used to treat focal cartilage lesions or degenerative joint diseases in children. It has been recently published successful results, with no reported complication, of a 6-year follow-up study in four children injected intra-articularly with autologous BM-derived MSCs [114].

However, it still remains to be determined whether the resulting cartilage formation was attributed to the *ex vivo* expanded MSC directly or via an indirect paracrine mechanism which led to inhibition of inflammatory responses or by stimulating the growth and/or activity of endogenous progenitors and chondrocytes. MSCs can differentiate into chondrocytes and fibrochondrocytes resulting in a mixture of cartilaginous fibrous and hypertrophic tissues with only a short-term clinical success, because they do not possess functional mechanical properties [112]. By contrast, MSCs derived from synovial tissue have been shown to enhance chondrogenic potential and reduce the level of hypertrophic differentiation in comparison with MSCs derived from BM [115, 116], probably due to the development of articular cartilage during embryogenesis. Several issues have to be clarified in order to perform a successful cartilage regeneration such as appropriate cell sources and scaffold, creating biomechanically suitable tissues and integrating to native ones [112].

## 5. Conclusions 

Osteogenic differentiation of hMSCs is tightly regulated by transcription factors that drive the type of tissue differentiation in hMSCs. The role of Runx2 and Wnt signaling has been elucidated in the past years both *in vitro* and *in vivo,* and growing evidence suggests that genetically modified MSC with these factors or with osteogenic factors such as BMP is useful to induce bone formation in pre-clinical models. The potential role of hMSCs in regenerative medicine is well known, and recent evidence suggests the potential use of hMSCs in bone regeneration in pediatric bone defects, particularly in the treatment of pseudarthrosis. Moreover, cell therapy for bone regeneration with lyophilized bone supplemented with platelet gel and BM hMSCs could be an adjuvant in the treatment of congenital pseudarthrosis of the tibial in pediatric orthopaedics. Further studies will be necessary to identify strategies for MSCs therapy for bone regeneration that take into account the characteristic of the tissue and permit to translate in a clinical setting the potential use of genetically modified MSCs.
